# A Chance for Benevolence and Philanthropy

**Published:** 1899-10

**Authors:** 


### A CHANCE FOR BENEVOLENCE AND PHILANTHROPY.

It is gratifying to note that a few medical journals are alive to the fact that it is the duty of the physician to do all in his power, both by pen and voice, to educate the people concerning the evil effects of alcohol, tobacco, and other drugs which have a deleterious and degenerating effect upon the human system.

The physician can have no nobler purpose than to devote his time to the prevention of disease. His field of labor in this direction is a broad one. There are opportunities for his services on every hand.

From the *Medical Brief* for May we quote the following concerning the evil effects of beer:

"Beer acts as a poison in the human system. It dulls the faculties, and when used habitually, tends to produce insanity in victims of the habit. The Germans, by long addiction, are able to indulge with less suffering than our countrymen, on the same principle that the Chinese coolie can subsist on rice alone with less digestive disturbances than other races can.

"There is little doubt, however, that the large consumption of beer in Germany is partially responsible, at least, for the decadence and degeneracy of the arts and sciences so manifest in the fatherland to-day. Science has gone to seed in Germany. An intolerable pedantry has taken its place, and when the intelligence of a nation loses its virility, it is time to look for the cause in the habits of the people.

"America can learn a lesson from the deplorable condition of Germany. The Anglo-Saxon cannot stand continual beer guzzling. By constitution and temperament he is less phlegmatic than his German brother. The fires of life, constantly fed by alcohol, would burn out in the more nervous organization of the American.

"Sumptuary laws will not check the evil of beer drinking.

"Prohibition would only arouse and strengthen a perverse determination to have it at any cost. Men cannot be coerced into doing right. Their minds must be convinced of the wisdom and justice of any measure, and then obedience can be secured without resorting to force.

"Men can be educated to understand that the drinking of beer, wine, brandy, whisky, or spirits of any kind is injurious to them. None of them contain nutritive principles. It is a marvel how such an idea ever came to be entertained. The medical profession is an influential body of men, enjoying peculiar facilities for teaching men truths of this kind. Doctors can do a world of good by calling attention to the wide-spreading damage done by alcohol.

"Alcohol pulls a man down and keeps him down. It absorbs his surplus earnings, so that he can never get ahead. It insidiously undermines his health. It robs him of pride, ambition, the power of conscientious accuracy and persevering application. It makes him careless, indifferent, spendthrift.

"Here is a mighty field where benevolence and philanthropy may labor for the regeneration of humanity, and physicians know better than any one else just how to set about it."—C. E. S., in *Modern Medicine*, July, 1899.

